# The impact of cardiopulmonary resuscitation (CPR) training on schoolchildren and their CPR knowledge, attitudes toward CPR, and willingness to help others and to perform CPR: mixed methods research design

**DOI:** 10.1186/s12889-020-09072-y

**Published:** 2020-06-12

**Authors:** Sanela Pivač, Primož Gradišek, Brigita Skela-Savič

**Affiliations:** 1 Angela Boškin Faculty of Health Care, Spodnji Plavž 3, SI-4270 Jesenice, Slovenia; 2 University Medical Center Ljubljana, Clinical Department of Anesthesiology and Intensive Therapy, and Medical Faculty, University of Ljubljana, Ljubljana, Slovenia

**Keywords:** Skills, Basic life support, Public health concern, Knowledge of schoolchildren

## Abstract

**Background:**

The benefits of cardiopulmonary resuscitation training for schoolchildren are well known, but the appropriate age for introducing training is still being discussed. This is a very important issue, since out-of-hospital cardiac arrest is a major public health concern. The objective of this study was to investigate the effects of implemented cardiopulmonary resuscitation training on the knowledge of schoolchildren in the last three grades of Slovenian elementary schools and theirs willingness, attitudes, and intentions toward helping others and performing cardiopulmonary resuscitation. The experience of training instructors was also explored.

**Methods:**

A mixed methods research design was employed, using a Separate Pre-Post Samples Design and focus groups. Research was conducted in 15 Slovenian public elementary schools offering cardiopulmonary resuscitation training. Focus groups included training instructors and developers. Data was collected with a structured questionnaire from April to June 2018 and analyzed using univariate and bivariate analyses. The three focus groups were convened in September and October 2018. Content analysis of the discussion transcriptions was conducted. The sample included 764 schoolchildren aged 12.5–14.5 years before cardiopulmonary resuscitation training and 566 schoolchildren after training. Three non-homogeneous focus groups included eight cardiopulmonary resuscitation instructors.

**Results:**

Significant progress in cardiopulmonary resuscitation knowledge was noted after training implementation, with the greatest progress seen in the youngest age group (mean age 12.5). The greatest increase after training was seen for the variables Attitude toward helping others (*p* = 0.001) and Self-confidence (*p* = 0.001). Analysis of the focus groups yielded two themes: (a) the effects of cardiopulmonary resuscitation training on schoolchildren, and (b) the systemic responsibility of the school system and professional bodies.

**Conclusions:**

Significant progress in schoolchildren’s cardiopulmonary resuscitation knowledge after training was established. Early introduction of training is recommended. Cardiopulmonary resuscitation knowledge raises awareness of the responsibility to help others and increases self-confidence to provide bystander cardiopulmonary resuscitation. It can be concluded that early cardiopulmonary resuscitation training for children is crucial. It should be a mandatory part of school curricula in those countries where cardiopulmonary resuscitation is not yet mandatory.

## Background

Out-of-hospital cardiac arrest (OHCA) is a major public health concern accounting for a substantial number of deaths worldwide. Each year, over 700,000 people in Europe and the USA suffer from OHCA. The survival to hospital discharge rate following OHCA remains low, ranging from 5 to 10% [1–3]. Bystander cardiopulmonary resuscitation (CPR) is crucial for improving the chances of survival of sudden cardiac arrest patients and their neurological outcomes [4]. Bystander CPR rates differ widely across European countries [3]. It is estimated that at least 15% of the population would have to be trained in CPR for a significant increase in survival rates after OHCA, but this cannot be achieved through voluntary trainings for the lay population alone; therefore, mandatory training of schoolchildren would be an important measure [5, 6].

Awareness of the importance of CPR must be raised in early childhood education [7], as CPR training improves the safety culture in schools and shifts the responsibility from adults to children, which could result in long-term structural changes [6].

The introduction of CPR training in schools has been advocated by the World Health Organization [5]. In Scandinavia, teaching schoolchildren CPR has increased lay bystander CPR rates, resulting in higher survival rates after OHCA. Besides that, the productivity of society raises and consequently, the costs of health care decline [8]. Teaching the importance of OHCA recognition and CPR skills should begin early, as schoolchildren have greater motivation and learn faster than adults and also maintain learning abilities [9, 10]. In some countries, CPR training for schoolchildren is already mandatory, in others it is being gradually introduced into curricula, with 2 hours of CPR training a year being recommended [11]. Nevertheless, the appropriate age to start CPR training remains controversial. If children learn CPR at a young age, they will not forget lifesaving skills, much like swimming or bike-riding skills [7]. In addition, children can serve as CPR multipliers as they may pass on the acquired awareness and CPR skills to family members and friends [12]. The advantages of early CPR teaching are reflected by attitude toward helping others, increased confidence in the positive outcome of resuscitation, internal motivation to help people requiring assistance, and development of empathy [13–17].

Change in behavior can be more effective if supported by the right structural measures, such as education and awareness raising; these findings are very relevant also in the field of public health [18]. Panchal et al. [19] claim that all interventions aiming at changing behavior are most effective if based in theory. In fact the social learning theory and the theory of planned behavior, both behavioral change theories, were developed as attempts to improve health education in order to ensure changes in behavior [20]. According to this theoretical grounding and the presentation of Intention-focused model of bystander CPR by Panchal et al. [19], we expect early CPR training to have an effect on the willingness, attitudes and intentions toward helping others and performing CPR. According to this model, it can be argued that changes in intention are followed by changes in behavior when individuals have the skills and ability to act. In their model, Panchal et al. predict changes in intention which lead to changes in behavior, possibly resulting in better CPR outcomes [19]. What is more, the model suggests that variables such as age, education, and gender lead to a change in behavior, although the authors [19] warn that not only these variables should be examined when it comes to evaluating CPR providers and their positive outcomes, but also variables which can be changed, consequently changing CPR providers’ intent and behavior. The intention-focused model by Panchal et al. is based in well-validated models, also in Ajzen’s theory of planned behavior (TPB) [21]. In his theory, Ajzen claims that “intentions are assumed to capture the motivational factors that influence a behavior; they are indications of how hard people are willing to try, of how much of an effort they are planning to exert, in order to perform the behavior” [21]. According to the two theories and our study, importantly based in findings in the fields of “effects of CPR training on the willingness to help others, increased self-confidence to perform CPR, motivation to help others in need, and the development of empathy”, we expect that early CPR training has an effect on willingness, attitudes and intentions toward helping others.

The effectiveness and outcome of CPR and automated external defibrillator (AED) training programs depend also on the instructor’s skills, knowledge and teaching tools (lecture, hands-only, instructional video, social networks) [16]. In elementary schools, the only healthcare professional is the school nurse who has a pivotal role as an instructor, coordinator, and policy advocate for CPR and AED training [22].

### Aim of the study

Our research aimed to investigate the effects of implemented CPR training on the willingness, attitudes, and intentions toward helping others and performing CPR and on CPR knowledge in schoolchildren of the last three grades of elementary school in Slovenia. In addition, we explored the training instructors’ experiences and opinions on the effects of implemented CPR training.

## Methods

### Design

A mixed methods research design was employed. The aim was to achieve a more comprehensive approach to the investigated topic, measurements, and the analysis and interpretation of findings [23]. A Separate Pre-Post Samples Design was conducted to obtain quantitative data and a focus groups research method was employed to obtain qualitative data. Both methods were combined in the interpretation stage.

### Setting and participants

#### Separate pre-post samples design

An intervention study using a ‘*Separate Pre-Post Samples Design’* is used when groups are being examined but where successive measurements are not sure to measure the same people [24], in which the dependent variable was measured before and again after a particular intervention was implemented [25, 26]. The intervention study included Slovenian elementary schools (15) which offered CPR training in April and May 2018. Information on which schools and classes actually offered CPR training in that period were obtained from the preventive medicine and health promotion services (part of community health centers); they informed us in advance of the planned CPR trainings. CPR training is not mandatory in Slovenian school curricula. Schools make the decision themselves whether or not to include CPR training in their programs. For research purposes, instructors covered the same educational content and used the same teaching methods; they followed the national CPR program based on the European Resuscitation Council guidelines. Thus, the intervention study included all schoolchildren of the seventh, eighth, and ninth grades of 15 elementary schools which provided CPR training in the observed timeframe. Before CPR training with the use of AED was offered, 893 elementary school children were invited to participate in the study and 764 responded (85.6% response rate). One to two months after CPR training, only those schoolchildren who received the training were invited to participate (*n* = 764), and 566 responded (74.1% response rate).

No significant differences were established in the sample structure prior to and after CPR training according to gender and age distribution, which means that both samples were uniform. The gender distribution in the sample is fairly equal, with just under 50% of participants being boys and just over 50% being girls. Children in the final three grades of elementary school were aged 12–15 years. The mean age of seventh-graders, eighth-graders and ninth-graders was 12.5, 13.5 and 14.5 years, respectively. Distribution of schoolchildren among the three grades was equal prior to and after training.

#### Focus group

Out of 12 invited experts, eight participated in focus groups: instructors and CPR program developers working as nursing professionals in primary health care. Six participants were women, two were men, and their average length of service was 21.4 years (SD = 12.8). One participant held a master’s degree in nursing, six were registered nurses, one was an emergency care assistant nurse. To start the debate, semi-structured guiding questions were used.

### Instrument

#### Separate pre-post samples design

In order to follow the intention-focused model for bystander CPR performance by Panchal et al. [19], we added a new variable termed ‘Knowledge’ to our study in order to determine whether schoolchildren’s newly obtained knowledge in CPR leads to a change in behavior which can be used to foster their willingness to help others, belief in own abilities, and self-confidence. Knowledge of CPR was measured using a structured questionnaire consisting of four sections with 27 nominal-level binary questions based on previous relevant research [5, 6, 15, 27, 28]. The sequence of items was designed according to the structure of the research problem. In developing the questionnaire, we followed the example of Tsang et al. [29]. The steps included determining the format in which the questionnaire will be administered (e.g. questionnaires for children should consider the cognitive stages of young people), the item format, and item development. Images were used to increase schoolchildren’s interest in filling out the questionnaire. Validity of the instrument was ensured by setting clear evaluation criteria and by making sure the questions were clearly formulated. Each correct answer was awarded one point; the point total was provided to compare results prior to and after CPR training. According to the final point count, respondents were categorized: excellent (15–13 points), very good (12–11 points), good (10–9 points), satisfactory (8–7 points), and unsatisfactory (6 points or less).

Willingness, attitudes and intentions toward helping others were gauged with an ordinal scale of opinions consisting of 22 items. Items were designed based on a literature review [6, 15, 17, 28]. The level of agreement was measured on a 5-point scale (1 = Strongly Disagree, 2 = Disagree, 3 = Neither Agree Nor Disagree, 4 = Agree, 5 = Strongly Agree). Content validity was assessed for the entire sample in which data was collected twice on the opinions on the willingness, attitudes, and intentions toward helping others, factor analysis was also conducted. For both data collections (prior to and after CPR training), we eliminated a few items with low factor loadings which is why differences exist in the structure of the first factor prior to and after the training, while items in the second factor remained the same (Table 2). Both instruments are comparable and result in two factors: Attitude toward helping others (Cronbach prior to training = 0.818; Cronbach after training = 0.592), and Self-confidence (Cronbach prior to training = 0.719; Cronbach after training = 0.554).

The willingness, attitudes and intentions toward helping others were assessed according to the level of knowledge, a prior-after was also conducted. Respondents were divided into two groups: the first included those with excellent knowledge (n1 = 45; n2 = 199) of CPR and the second those with less than excellent CPR knowledge (n1 = 718; n2 = 366).

The demographic part of the questionnaire included questions on age, grade, month and year of birth.

#### Pilot study

Two pilot studies were carried out. The first one was conducted prior to CPR training and included 66 schoolchildren. For 26 items Cronbach’s alpha was 0.78. We excluded four items with low reliability. Additional instructions were provided for nominal-level binary questions used to test schoolchildren’s CPR knowledge in order to clarify the question and the possible number of answers. With these improvements to the questionnaire, the second pilot study was carried out after CPR training (*n* = 64). Cronbach’s alpha for 22 items on the willingness, attitudes and intentions toward helping others was 0.81 for 63 schoolchildren. Face and content validity was established for the first pilot study. After filling them out, respondents reported that the questionnaires were understandable, but somewhat too long. Participants did not indicate any possible unintelligibility of items on CPR knowledge.

#### Focus group instrument

Semi-structured guiding questions were used for the focus groups and expanded as the discussion developed. Research reliability was ensured by following the prescribed methodology: the appropriate number of focus groups (*n* = 3) and the number of participants in each group (*n* = 8), which ensured data saturation in the third focus group.

### Data collection

Separate Pre-Post Samples Design was conducted twice from April to June 2018: prior to CPR training and one to two months following the training (May, June 2018).

Focus groups were convened after the implemented CPR training, in September and October 2018. The focus groups were led by a moderator employing a semi-structured questionnaire. All selected research participants received an invitation with a brief description of the research, general topics, and partial results of the quantitative research. The average duration of each focus group was 60 min. Discussions were recorded with participants’ written consent.

### Data analysis

Data was analyzed using IBM software SPSS Statistics v. 22.0. Descriptive statistics was used to present the results of CPR knowledge test and the answers to the questions on the willingness, attitudes and intentions toward helping others, chi-square test was used to analyze the differences in the percentage of correct answers on the knowledge test prior to and after CPR training. For establishing the differences in the level of knowledge and the willingness, attitudes and intentions toward helping others prior to and after CPR training, a t-test for comparing two samples was employed. Exploratory factor analysis (EFA) was performed prior to (N1 = 737) and after (N2 = 565) CPR training after first confirming that data were suitable for factor analysis. Bartlett’s Test of Sphericity was highly significant (*p* < 0.001) and the Kaiser-Meyer-Olkin (KMO) measure of sampling adequacy supported the factorability of the matrix (prior to training = 0.880; after training = 0.729). Principal components analysis (PCA) was used to assess the number of factors to be extracted, followed by oblique rotation of factors using Oblimin rotation. The number of factors to be retained was supported using Horn’s parallel analysis, the factors were extracted using the PAF method (rotation method: Oblimin).

For qualitative data, the method of thematic content analysis was employed. All recordings were transcribed verbatim and the texts were read several times. After coding units were identified, coding was conducted and categories and key themes were defined. Each focus group was ascribed a corresponding code. The nominal identity of a transcription was lost while the traceability of content was ensured.

## Results

### CPR knowledge and the willingness, attitudes and intentions toward helping others prior to and after CPR training

Significant progress in knowledge was noted in most CPR-related procedures one or two months after the training. The greatest progress was in placing AED electrodes in the right positions, and in the chest compression rate and depth (all *p* = 0.001). The mean score for the variable ‘Knowledge’ was 9.5 (SD = 2.0) prior to and 11.5 (SD = 2.0) after CPR training (*p* = 0.001). However, training did not increase the knowledge of other AED-related procedures like checking responsiveness and breathing before using AED (Table 1).

**Table 1:** CPR knowledge of schoolchildren (N1 = 764 / N2 = 566) prior to and after CPR training

		Assessment				chi-square statistic (*p*-value)
		prior to training (N1)		after training N2)		
		n	%	n	%	
Phone number for medical emergencies in Slovenia is 112.	Wrong answer	54	7.1	6	1.1	27.244 (0.001)
	Right answer	710	92.9	560	98.9	
I have to tell the healthcare dispatcher my location.	Wrong answer	80	10.5	35	6.2	7.566 (0.006)
	Right answer	684	89.5	531	93.8	
I have to tell the healthcare dispatcher what happened.	Wrong answer	95	12.4	50	8.8	4.339 (0.037)
	Right answer	669	87.6	516	91.2	
I have to tell the healthcare dispatcher how many people need assistance.	Wrong answer	190	24.9	108	19.1	6.265 (0.012)
	Right answer	574	75.1	458	80.9	
An unconscious person must be rolled onto their side.	Wrong answer	206	27.0	85	15.0	27.144 (0.001)
	Right answer	558	73.0	481	85.0	
An automated external defibrillator is an electronic device capable of recognizing if a person’s heart is still beating.	Wrong answer	357	46.7	188	33.2	25.544 (0.001)
	Right answer	407	53.3	378	66.8	
When a person is showing no signs of life or is unresponsive, I have to secure the scene, locate possible victims, and dial 112.	Wrong answer	275	36.0	139	24.6	19.836 (0.001)
	Right answer	489	64.0	427	75.4	
Breathing is checked by tilting the victim’s head back, listening for breathing sounds over their mouth, and looking to see if the chest is rising or falling.	Wrong answer	141	18.5	52	9.2	22.513 (0.001)
	Right answer	623	81.5	514	90.8	
The chest should be compressed 5 cm in depth.	Wrong answer	310	40.6	124	21.9	51.541 (0.001)
	Right answer	454	59.4	442	78.1	
100–120 chest compressions should be performed per minute.	Wrong answer	583	76.3	203	35.9	220.004 (0.001)
	Right answer	181	23.7	363	64.1	
The image of a chest has different positions marked; please indicate where hands must be placed when compressing the chest.	Wrong answer	275	36.0	123	21.7	31.543 (0.001)
	Right answer	489	64.0	443	78.3	
The image of a chest has different positions marked; please indicate where AED electrodes must be placed.	Wrong answer	603	78.9	173	30.6	312.886 (0.001)
	Right answer	161	21.1	393	69.4	
Prior to using an AED, check the victim’s responsiveness and breathing, then switch on AED and follow the voice instructions.	Wrong answer	123	16.1	71	12.5	3.299 (0.069)
	Right answer	641	83.9	495	87.5	
When using an AED, dial 112 and wait for instructions on how to use the device.	Wrong answer	492	64.4	354	62.5	0.483 (0.487)
	Right answer	272	35.6	212	37.5	
Start resuscitating a drowning victim by checking their breathing, give them five initial breaths, and then follow with chest compressions.	Wrong answer	438	57.3	254	44.9	20.203 (0.001)
	Right answer	326	42.7	312	55.1	

Legend: *AED* Automated external defibrillator, *N1* Number of schoolchildren respondents prior to CPR training, *N2* Number of participating schoolchildren after CPR training, *n* Number, *%* Percentage, *p* Borderline statistical significance of 0.05 and less

Prior to the training, ninth-graders (average age of 14.5 years) had the highest level of knowledge (M = 10.0; SD = 2.03), followed by seventh-graders (average age of 12.5 years) (M = 8.96; SD = 2.19) and eighth-graders (average age of 13.5 years) (M = 9.37; SD = 1.98) being behind by a point on average. The differences in CPR knowledge between age groups were significant (*p* = 0.001). After the training, the level of knowledge increased in seventh-graders (M = 11.62; SD = 2.04), eighth-graders (M = 11.43; SD = 2.10), and ninth-graders (M = 11.52; SD = 2.00). The increase in the level of knowledge for each age group when comparing the results prior to and after the training was significant (*p* < 0.05). After CPR training, all age groups had a comparable level of CPR knowledge. However, seventh-graders achieved the largest absolute difference in knowledge level increase (from 8.96 to 11.62 points on average).

The total means for items gauging the willingness, attitudes and intentions toward helping others ranged between 3.39 and 4.76 prior to training and 3.92 and 4.73 after training. Factor analysis of these items revealed two constructs based on 17 items for F1–Attitude toward helping others and for F2–Self-confidence (loadings > 0.3). Variance was explained in 30.96% prior to CPR training: F1–Attitude toward helping others explained 24.14% of the variance (*n* = 737; α = 0.818), F2–Self-confidence explained 6.82% of the variance (n = 737; α = 0.719). In the second data collection after CPR training, the total variance explained was 22.08%: F1–Attitude toward helping others explained 14.49% of the variance (*n* = 565; α = 0.592), and F2–Self-confidence explained 3.87% of the variance (n = 565; α = 0.554). The distribution of items is shown in Table 2.

**Table 2:** Prior to and after CPR training willingness, attitudes, intentions toward helping others and performing CPR in schoolchildren (N1 = 764 / N2 = 566)

Items	Prior to training			After training			Factor loadings prior to (*n* = 737) and after training (*n* = 565)			
	n	M	SD	n	M	SD	F1 prior F1 after		F2 prior F2 after	
If someone fell down in front of me, I would help them. (1)*	761	4.49	0.77	565	4.61	0.55	0.416	0.569		
If my friend lost consciousness, I would help them. (2)	762	4.76	0.54	565	4.73	0.48	0.439	0.642		
I am willing to help others because I would also expect help when in need. (3)	760	4.49	0.71	565	4.48	0.60	0.576	0.524		
I help because I can recognize a person who is not showing signs of life. (4)*	757	3.89	0.87	565	4.28	0.56	0.433			
I like to help others when they are in need. (5)*	762	4.29	0.79	565	4.44	0.56	0.655	0.338		
I notice when my friends and classmates need help. (6)	760	4.18	0.85	565	4.26	0.69	0.542			
When I notice that someone needs help, I ask myself how I can help them. (8)*	756	4.01	0.98	565	4.33	0.65				
If someone fell down in front of me, I would call for help immediately. (9)	760	4.36	0.87	565	4.41	0.65	0.306			
If I noticed a group of people only observing a victim not showing signs of life, I would start providing help immediately. (10)	761	4.21	0.89	565	4.22	0.74	0.491			
I am aware that by helping a victim not showing signs of life, I can save their life. (11)	761	4.42	0.81	565	4.43	0.68	0.333			
If someone fell down in front of me and showed no signs of life, I would start CPR. (13)*	763	3.84	1.10	565	4.17	0.79			0.444	0.302
If I noticed a victim who was not moving or showing signs of life, I would begin CPR immediately, because I am not afraid of injuring them. (14)*	762	3.39	1.18	565	3.96	0.84			0.734	0.444
If I noticed a victim who was not moving or showing signs of life, I would begin CPR immediately, because I believe in myself. (15)*	759	3.43	1.09	565	3.99	0.78			0.676	0.539
When friends ask for my help, I don’t hesitate and help them right away. (17)	760	4.29	0.78	565	4.25	0.80	0.511			
I feel for classmates and friends who are very ill or were struck by misfortune. (18)	759	4.34	0.82	565	4.33	0.69	0.601			
I think one of the best things is being able to help others. (19)	757	4.14	0.88	565	4.19	0.77	0.573	0.332		
Helping others gives me satisfaction. (20)	759	4.27	0.82	565	4.32	0.71	0.585			
I would dare to provide CPR before receiving training. (21)*	761	3.42	1.23	565	3.92	0.94			0.575	0.541
I would dare to provide CPR after receiving training. (22)*	759	4.06	0.98	565	4.31	0.64			0.367	0.395

Legend: *CPR* Cardiopulmonary resuscitation, *M* Mean on a 5-point scale (1 – Strongly Disagree / 5 – Strongly Agree), *N1* Number of schoolchildren respondents prior to CPR training, *N2* Number of schoolchildren respondents after CPR training, *n* Number of answers, *SD* Standard deviation, *F1* Attitude toward helping others; F2 = Self-confidence; *Differences in mean values between the first and second data collections are statistically significant (*p* < 0.05)

A statistically significant difference and improvement after CPR training were established for both variables: Attitude toward helping others (*p* = 0.001) and Self-confidence (p = 0.001) (Table 3). Schoolchildren with excellent CPR knowledge displayed, both prior to and after CPR training, greater willingness, better attitudes and greater intention toward helping others. The greatest increase after the training was seen in Attitude toward helping others (p = 0.001); what is more, the number of schoolchildren who received an excellent grade after the training increased (Table 4).

**Table 3:** A comparison of factor analysis components prior to (N1 = 764) and after CPR training (N2 = 566)

	Before CPR training			After CPR training			t statistics (p-value) (before – after)
	n	M	SD	n	M	SD	
Attitude toward helping others *	763	4,31	0,46	565	4,48	0,35	-7291 (0,001)
Self-confidence *	763	3,63	0,77	565	4,07	0,48	−12,785 (0,001)

Legend: *N1* Number of schoolchildren respondents prior to CPR training, *N2* Number of schoolchildren respondents after CPR training, *n* Number of answers, *M* Mean, *SD* Standard deviation; The difference between components prior to and after CPR training is statistically significant (*p* < 0.05)

**Table 4:** A comparison of factor components in schoolchildren (N1 = 764 / N2 = 566) with excellent CPR knowledge vs. in those with lower level of CPR knowledge

Grouping according to number of points achieved		Prior to training (*n* = 763)				After CPR training (n = 565)			
		n1	Mean	Std. Deviation	t statistics (*p*-value)	n2	Mean	Std. Deviation	t statistics (p-value)
Attitude toward helping others *	Lower level of CPR knowledge	718	4,30	0,46	− 3876 (0,001)	366	4,45	0,35	− 2165 (0,031)
	Excellent CPR knowledge	45	4,55	0,41		199	4,52	0,34	
Self-confidence *	Lower level of CPR knowledge	718	3,60	0,77	− 3403 (0,001)	366	4,08	0,48	0,561 (0,575)
	Excellent CPR knowledge	45	4,00	0,71		199	4,05	0,49	

Legend: *N1* Number of schoolchildren respondents prior to CPR training, *N2* Number of schoolchildren respondents after CPR training, *n* Number of answers, *M* Mean, *SD* Standard deviation; t-test; *Difference is statistically significant (*p* < 0.05*)*

### Instructors’ perspectives on and experiences with CPR training

Following text analysis, two main themes were developed: (a) Effects of CPR training on schoolchildren, and (b) Systemic responsibility of the school system and professional affiliations. Randomly selected examples of instructors’ comments, positive and negative views on CPR training, and suggestions for school curriculum developers and responsible authorities are presented in Table 5.

**Table 5:** Suggestions from instructors

THEMES	SELECTED EXAMPLES OF COMMENTS	POSITIVE EFFECTS OF CPR TRAINING	NEGATIVE EFFECTS OF CPR TRAINING	SUGGESTIONS FOR SCHOOL CURRICULUM DEVELOPERS AND RESPONSIBLE BODIES
*Effects of CPR training on schoolchildren*	*(F3/U1): “They’re mainly willing to help others; they notice when a friend needs help.”*	Increased prosocial behavior.	Resuscitation topics are not mandatory in the curriculum of Slovenian elementary schools.	CPR topics would have to become a mandatory part of school curricula at the national level.
	*(F1/U1): “When a kid had an episode of hypoglycemia, his classmate called for help”.*	Raising awareness of the responsibility to help others.		CPR training has to begin early, topics are adapted to the age of schoolchildren.
	*(F2/U1): “After the training they’re kind of more confident, I think they’re also happy they can try chest compressions and talk to each other about that, it’s kind of an internal satisfaction that I sense in the workshops.”*		More difficult entry of instructors into the school setting.	Trainings have to be organized in small groups.
		Positive effect on emotional development.		Setting minimum criteria for instructors by professional interdisciplinary societies: required certification, the need to follow ERC recommendations on CPR, appropriate teaching methods employed by instructors, provision of appropriate equipment and tools for the implementation of CPR training, CPR refreshment courses, both for instructors and schoolchildren.
	*(F1/U1): “It’s definitely an investment in our children, so to say, to teach them early enough about resuscitation.”*	Less fear.	Instructors have different levels of knowledge on CPR training.	
		Increased confidence.		
	*(F1/U3): “The point of the workshops is that you’re not scared of consequences in an actual situation when someone falls down, so that most kids aren’t scared …*”	Satisfaction and independence of schoolchildren.		
		Long-term memory of storing information on resuscitation procedures.		
*Systemic responsibility of the school system and professional affiliations*	*(F2/U2): “I think it’s important that topics on resuscitation are included in the school curriculum as mandatory, it has to be done at the national level and everything connected to it” and (F3/U1):* “*… .sure, topics have to be adapted for the age group … ..but even first-graders can bring an AED, position the stickers and switch it on. It’s been proven that even little kids can help that way. Even in daycare, like if they watch a video of how compressions are done.”*			
	*(F1/U2):* “*… someone would have to assume the overall management of the instructors who would then be giving trainings, just like the section does, that’s how it should be. I think it’s in the best interest that trainings are the same across Slovenia, not that we have to get creative about how to incorporate guidelines.”*			

Legend: *F1–3/U1–3* F1–3 = focus group number, *U1–3* Identification of focus group participant

## Discussion

The effects of implementing CPR training with the use of AED on the knowledge of schoolchildren and their willingness, attitudes and intentions toward helping others was investigated. The study demonstrated an improvement in theoretical knowledge after CPR training. The provided training clearly increased schoolchildren’s willingness, and improved their attitudes and intentions toward helping others. Our findings indicate that CPR training alone can raise not only children’s level of CPR knowledge, but also increase their willingness, and improve their attitudes and intentions toward helping others.

Specifically, children showed an improvement after training in theoretical knowledge on the question of which information has to be provided to paramedics in case a person is found unconscious and the correct position of an unconscious victim. Virtually all schoolchildren were familiar with the emergency telephone number in Slovenia, a result is comparable to the findings of previous studies [10], which underlies the positive effects of trainings. The greatest progress in 566 schoolchildren was seen in the knowledge of actions to be taken with an unconsciousness person, the placement of AED electrodes in correct positions, and the frequency and depth of chest compressions. Knowledge on the latter point after training was similar to findings reported from other studies [10]. Most of the schoolchildren in our research knew the right answer on how to use an AED; however, they were not as familiar with the fact that the emergency telephone number can also be dialed to receive instructions on its use. Schoolchildren are capable of determining whether the victim is conscious, calling for help, providing relevant information on the victim, and using AED [30]. Our research showed some progress on the correct CPR of a drowning victim, but the percentage of incorrect answers still remains high. Therefore, CPR instructions to schoolchildren should be simple and delivered uniformly for all causes of OHCA.

Our results revealed that the level of schoolchildren’s theoretical knowledge on CPR was higher after CPR training and that the level of knowledge was retained one to two months after training. Similarly, other researchers found not only greater knowledge after CPR training, but also its retention [27, 31, 32]. Prior to CPR training, the level of CPR knowledge was highest in children with a mean age of 14.5 years, while children with mean ages of 12.5 and 13.5 years were less successful. After the implemented CPR training, the level of knowledge increased in all age groups. The greatest progress was seen in the youngest age group (mean age 12.5), because children in this group had to gain the most knowledge to be on par with their older peers. Therefore, we support early introduction of CPR training. Other authors have made similar recommendations [16, 30, 32, 33]. What is more, younger schoolchildren have a greater capacity to learn the practical aspects of resuscitation compared to older children [32]. In Germany, research showed that the ability to implement practical CPR interventions among 10-year-olds was the same as among 13-year-olds [15]. However, schoolchildren aged 13 or more have greater theoretical knowledge [15, 32, 34]. Considering the assessed effectiveness of CPR training in schoolchildren, some advocate the introduction of CPR training between the ages of 10 and 11. In this age group, schoolchildren have the necessary intellectual capacity and, on average, an appropriate body weight to provide effective chest compressions [15, 16]. Moreover, younger schoolchildren know how to place electrodes on the chest fast and effectively, while also ensuring safety beforehand [16, 32].

In the second part of our research, we compared the level of willingness, attitudes and intentions toward helping others prior to and after CPR training. After training, participants showed a significant increase in the attitude toward helping others and self-confidence to help others. In children of the final three grades of elementary school, increased CPR knowledge led to greater willingness, and improved attitudes and intentions toward helping others. Similar conclusions were also reached by previous researchers [13, 31]. As in our research findings, previous studies showed that CPR training boosts the confidence of schoolchildren [13]. Our study also showed that schoolchildren with excellent CPR knowledge express a greater willingness to help others. The percentage of such schoolchildren increased after CPR training.

CPR training for schoolchildren significantly increased their self-worth and moral responsibility towards themselves and the people around them [31]. CPR training reduced the fear of making a mistake in persons providing assistance to cardiac arrest victims, raised self-confidence, and fostered children’s attitude toward helping others [15].

Our research results also showed that, after CPR training, the percentages of those willing to help others increased. Similar studies stress the importance of early learning and implementation of training in intervals, because they believe that recurring trainings increase the willingness, and improve the attitudes and intentions toward helping others of schoolchildren [16]. Similarly, previous studies demonstrated that early learning of helping others makes schoolchildren better understand the importance of helping others and evolves their capacity for expressing empathy [35].

We were also interested in the instructors’ experiences and opinions on the effects of the conducted CPR training. Analysis of the focus groups suggests training in CPR has to start at an early stage, with the content and teaching tools adjusted to the children’s age.

The European Resuscitation Council Initiative and World Health Organization Statement endorse making CPR training a mandatory part of school curricula, as this would have a significant influence on the public health issue related to sudden cardiac arrest [5, 11, 36]. Focus group results showed obstacles for introducing CPR training in Slovenian schools, most notedly that CPR is at the moment an elective subject, not part of the mandatory school curricula. Furthermore, current instructors have been found to have different levels of knowledge and skills, highlighting the need for setting minimum criteria for certified CPR instructors. Closer cooperation with professional associations, which should set the minimum criteria for the implementation of trainings and for the potential instructors, is also required. According to the focus group members, these minimum criteria are: introduction of mandatory CPR training in school curricula, appropriate qualifications of instructors who must follow ERC recommendations on CPR, appropriate teaching methods employed by instructors, appropriate equipment and tools, the ability to adapt the content according to children’s age, trainings in small groups, refreshment of knowledge both for instructors and schoolchildren. Support and guidance of instructors and other adults involved is crucial for the development of a responsible and emphatic approach towards teaching schoolchildren CPR [37].

We see the main contribution of this research as providing policy/decision-makers with a solid theoretical foundation to include CPR training as a mandatory part of school curricula. The results revealed positive effects of training schoolchildren in CPR, making it very important to include CPR training in school curricula early enough. We found that children aged 12.5 years made the most progress in CPR knowledge.

It can be concluded that early CPR training for children is crucial and should be introduced as a mandatory part of school curricula in those countries where CPR is not yet mandatory.

### Limitations and strengths

A strength of the presented research is the sample size. The validity of conclusions could be increased if the methodology included an assessment of practical CPR skills. The study only investigated schoolchildren’s theoretical knowledge. We are aware that testing only theoretical knowledge is not the only option, so we suggest future studies focus on the effects of practical training on CPR with the use of AED on schoolchildren’s knowledge. The limitation of the study is that it was based on a non-standardized, self-developed questionnaire using ad hoc measures which was not yet psychometrically tested for reliability, validity, discriminant validity and sensitivity. The limitation is also the fact that the groups at the pre-post test were non-equivalent, and this could bias the results. We cannot match individual participant responses from pre to post, we can only look at the change in average mean from pre-test to post-test. Thus, we followed the progress at the group level, not at the individual level. According to factor analysis results, we believe qualitative research should be conducted among the target population on the understanding of the importance of CPR and on schoolchildren’s understanding of their role in the importance of helping others. Such research would provide the information necessary to improve the validity and reliability of our instrument.

The strength of correlations in the quantitative part of the research is low due to the proportions of factorial analysis variance explained, so these findings represent the basis for further improvements of the research instrument. The third focus group sample size was small since only two of the four invited participants responded by the given date. However, the third focus group was not cancelled due to ethical principles; it was implemented and in-depth conclusions were made.

## Conclusions

The research findings provide the basis for decision-makers to introduce CPR training as mandatory part of school curricula. We must also be aware that the endeavors for CPR topics to become a mandatory part of school programs can only be successful by employing a comprehensive, responsible approach and with the awareness of social responsibility. Nevertheless, it is very important to involve the CPR training into curricula. The youngest age group (12.5 years) made the most progress in CPR knowledge.

Results of the quantitative and qualitative research clearly demonstrate the positive effects of training schoolchildren in CPR. We found that the level of CPR knowledge correlated with the willingness, attitudes and intentions toward helping others important factors in schoolchildren’s social development and the development of their values, opinions, and beliefs. This research gives an important contribution to public health policy. It provides criteria for implementation of training schoolchildren in CPR, which is one of crucial factors of higher survival rate at cardiac arrest.

Our study findings are important for decision-makers and developers of school curricula who are responsible for making CPR training a mandatory part of school curricula. CPR training should be conducted continuously, for several years from the time schoolchildren are 12, and should be part of the mandatory school curricula. By doing so, we believe that CPR outcomes in cardiac arrest victims could significantly improve in the future. What is more, the necessary equipment, teaching materials, and teaching aids should be available to CPR training instructors, both for theoretical and practical training, as they are indispensable for efficient learning. This equipment should be provided by the institutions responsible for offering CPR training with the use of AED.

## Data Availability

The datasets used and/or analyzed during the current study are available from the corresponding author on reasonable request.

## Abbreviations

CPR: Cardiopulmonary resuscitation
OHCA: Out-of-hospital cardiac arrest
AED: Automated external defibrillator

## References

[1] Berdowski J, Berg RA, Tijssen JG, Koster RW (2010). Global incidences of out-of-hospital cardiac arrest and survival rates: systematic review of 67 prospective studies. Resuscitation..

[2] Nishiyama C, Brown SP, May S, Iwami T, Koster RW, Beesems SG (2014). Apples to apples or apples to oranges? International variation in reporting of process and outcome of care for out-of-hospital cardiac arrest. Resuscitation..

[3] Gräsner JT, Lefering R, Koster RW, Masterson S, Böttiger BW, Herlitz J (2016). EuReCa ONE-27 nations, ONE Europe, ONE registry: a prospective one month analysis of out-of-hospital cardiac arrest outcomes in 27 countries in Europe. Resuscitation..

[4] Geri G, Fahrenbruch C, Meischke H, Painter I, White L, Rea TD (2017). Effects of bystander CPR following out-of-hospital cardiac arrest on hospital costs and long-term survival. Resuscitation..

[5] Böttiger BW, Van Aken H (2015). Kids save lives--training school children in cardiopulmonary resuscitation worldwide is now endorsed by the World Health Organization (WHO). Resuscitation..

[6] Calicchia S, Cangiano G, Capanna S, De Rosa M, Papaleo B (2016). Teaching life-saving manoeuvres in primary school. Biomed Res Int.

[7] De Buck E, Van Remoortel H, Dieltjens T, Verstraetena H, Claryssea M, Moens O (2015). Evidence-based educational pathway for the integration of first aid training in school curricula. Resuscitation..

[8] Wissenberg M, Lippert FK, Folke F, Weeke P, Hansen CM, Christensen EF (2013). Association of national initiatives to improve cardiac arrest management with rates of bystander intervention and patient survival after out-of-hospital cardiac arrest. JAMA..

[9] Markenson D, Ferguson J, Chameides L, Cassan P, Chung K, Epstein J (2010). Part 17: first aid: 2010 American Heart Association and American red cross guidelines for first aid. Circulation..

[10] Banfai B, Pek E, Pandur A, Csonka H, Betlehem J (2017). The year of first aid’: effectiveness of a 3-day first aid programme for 7-14-year-old primary schoolchildren. Emerg Med J.

[11] Böttiger BW, Semeraro F, Altemeyer KH, Breckwoldt J, Kreimeier U, Rücker G (2017). Kids save lives: school children education in resuscitation for Europe and the world. Eur J Anaesthesiol.

[12] Greif R, Lockey AS, Conaghan P, Lippert A, De Vries W, Monsieurs KG (2015). European resuscitation council guidelines for resuscitation 2015. Section 10. Education and implementation of resuscitation. Resuscitation.

[13] Maconochie I, Bingham B, Simpson S (2007). Teaching children basic life support skills. BMJ..

[14] Patsaki A, Pantazopoulos I, Dontas I, Passali C, Papadimitriou L, Xanthos T (2012). Evaluation of Greek high school teachers' knowledge in basic life support, automated external defibrillation, and foreign body airway obstruction: implications for nursing interventions. J Emerg Nurs.

[15] Bohn A, Van Aken HK, Möllhoff T, Wienzek H, Kimmeyer P, Wild E (2012). Teaching resuscitation in schools: annual tuition by trained teachers is effective starting at age 10. A four-year prospective cohort study. Resuscitation..

[16] Plant N, Taylor K (2013). How best to teach CPR to schoolchildren: a systematic review. Resuscitation..

[17] Decety J, Bartal IBA, Uzefovsky F, Knafo-Noam A (2016). Empathy as a driver of prosocial behaviour: highly conserved neurobehavioural mechanisms across species. Philos Trans R Soc Lond B Biol Sci.

[18] Laverack G (2017). The challenge of behaviour change and health promotion. Challenges..

[19] Panchal AR, Fishman J, Camp-Rogers T, Starodub R, Merchant RM (2015). An “intention-focused” paradigm for improving bystander CPR performance. Resuscitation..

[20] Reed M, Lloyd B (2018). Health Psychology.

[21] Ajzen I (1991). The theory of planned behavior. Organ Behav Hum Decis Process.

[22] Boudreaux S, Broussard L (2012). Sudden cardiac arrest in schools: the role of the school nurse in AED program implementation. Issues Compr Pediatr Nurs.

[23] Polit DF, Beck CT (2018). Essentials of nursing research: appraising evidence for nursing practice.

[24] Trochim WM, Donnely JP, Arora K (2015). Research methods: the essential knowledge base.

[25] Price PC, Rajiv J, Chiang I-C A, Leighton DC, Cuttler C (2017). Research methods in psychology.

[26] Thiese MS (2014). Observational and interventional study design types: an overview. Biochem Med.

[27] Meissner TM, Kloppe C, Hanefeld C (2012). Basic life support skills of high school students before and after cardiopulmonary resuscitation training: a longitudinal investigation. Scand J Trauma Resusc Emerg Med.

[28] Petrić J, Malički M, Marković D, Meštrović J (2013). Students’ and parents’ attitudes toward basic life support training in primary schools. Croat Med J.

[29] Tsang S, Royse C, Terkawi A (2017). Guidelines for developing, translating, and validating a questionnaire in perioperative and pain medicine. Saudi J Anaesth.

[30] Bohn A, Lukas RP, Breckwoldt J, Böttiger BW, Van Aken H (2015). ‘Kids save lives’: why school children should train in cardiopulmonary resuscitation. Curr Opin Crit Care.

[31] Naqvi S, Siddiqi R, Hussain SA, Batool H, Arshad H (2011). School children training for basic life support. J Coll Physicians Surg Pak.

[32] Lukas RP, Van Aken H, Mölhoff T, Weber T, Rammert M, Wild E (2016). Kids save lives: a six-year longitudinal study of schoolchildren learning cardiopulmonary resuscitation: who should do the teaching and will the effects last?. Resuscitation..

[33] Cave DM, Aufderheide TP, Beeson J, Ellison A, Gregory A, Hazinski MF (2011). Importance and implementation of training in cardiopulmonary resuscitation and automated external defibrillation in schools: a science advisory from the American Heart Association. Circulation..

[34] Wingen S, Schroeder DC, Ecker H, Steinhauser S, Altin S, Stock S (2018). Self-confidence and level of knowledge after cardiopulmonary resuscitation training in 14 to 18-year-old schoolchildren: a randomised-interventional controlled study in secondary schools in Germany. Eur J Anaesthesiol.

[35] Bollig G, Wahl HA, Svendsen MV (2009). Primary school children are able to perform basic life-saving first aid measures. Resuscitation..

[36] Link MS (2007). CPR for kids: please try this at home. J Watch Cardiol.

[37] Matchim Y, Kongsuwan W (2015). Thai nursing students' experiences when attending real life situations involving cardiac life support: a phenomenological study. Nurse Educ Today.

